# Robust detection and refinement of saliency identification

**DOI:** 10.1038/s41598-024-61105-3

**Published:** 2024-05-14

**Authors:** Abram W. Makram, Nancy M. Salem, Mohamed T. El-Wakad, Walid Al-Atabany

**Affiliations:** 1 Biomedical Engineering Department, Faculty of Engineering, Helwan University, Helwan, Egypt; 2 Faculty of Engineering and Technology, Future University, Cairo, Egypt; 3 Information Technology and Computer Science School, Nile University, Giza, Egypt

**Keywords:** Engineering, Biomedical engineering

## Abstract

Salient object detection is an increasingly popular topic in the computer vision field, particularly for images with complex backgrounds and diverse object parts. Background information is an essential factor in detecting salient objects. This paper suggests a robust and effective methodology for salient object detection. This method involves two main stages. The first stage is to produce a saliency detection map based on the dense and sparse reconstruction of image regions using a refined background dictionary. The refined background dictionary uses a boundary conductivity measurement to exclude salient object regions near the image's boundary from a background dictionary. In the second stage, the CascadePSP network is integrated to refine and correct the local boundaries of the saliency mask to highlight saliency objects more uniformly. Using six evaluation indexes, experimental outcomes conducted on three datasets show that the proposed approach performs effectively compared to the state-of-the-art methods in salient object detection, particularly in identifying the challenging salient objects located near the image's boundary. These results demonstrate the potential of the proposed framework for various computer vision applications.

## Introduction

Salient object detection is an image analysis technique that intends to automatically identify the visually significant regions in an image. Inspiration for salient object detection comes from the human visual system, which is able to quickly and efficiently recognize important objects in a visual scene. Therefore, the main contribution of salient object detection appears in assistive technologies for individuals with visual impairments^1^, including those using retinal prostheses to overcome the limited resolution of current retinal prostheses by identifying the most visually important areas in a scene and enhancing their visibility for retinal prosthesis users^2^. Also, Salient object detection has numerous applications in the fields of image processing and computer vision, including object recognition^3^, image editing, compression^4^, autonomous driving^5^, and medical imaging^6^.

Recently, deep learning has achieved a breakthrough in the saliency detection field, but there are some limitations mainly related to the high computational power required and complex architectures. Also, they may fail to preserve the object boundary and edges. So, Traditional saliency detection provides a lifeline for applications with limited data and resources as they generally need less computational power and memory. Therefore, Traditional methods are still an attractive research area.

Inspired by the biological visual attention mechanism, traditional saliency detection methods typically rely on contrast analysis of low-level features. The primary system for saliency detection, introduced by Itti et al.^7^, utilizes a contrast-based model that specifically employs center-to-surround differences for multiscale low-level features. These methods exhibit some challenges related to (1) sensitivity to low-level features, where minor color variation can lead to inaccuracies, and (2) the lack of high-level contextual understanding. Therefore, incorporating background priors can alleviate these issues. This inclusion allows for a better understanding of the scene, leading to improved accuracy in saliency detection and a better balance between recognizing the global context and capturing local details in the image. The background priors are built on the observation that the areas of an image closer to the image border are more probable to belong to the background. On the other hand, using the image border areas directly as a background dictionary to find the salient object may fail to identify the near-boundary-salient object as the background dictionary includes parts of this object. Therefore, refining the background dictionary is a critical issue.

**Figure 1:**
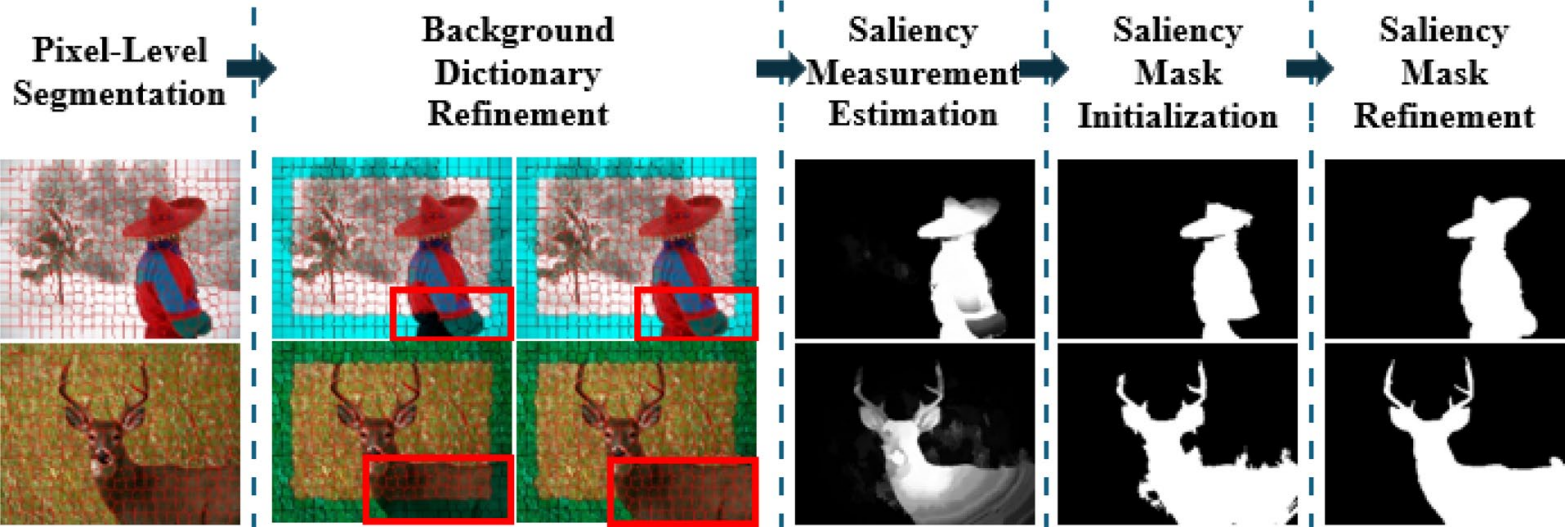
Pipeline of the proposed Method. Images represent the output of each stage (Sections "Pixel-Level Image Segmentation", "Background dictionary refinement", "Saliency measurement estimation", "Saliency mask initialization", "Saliency mask refinement"). The input of the pipeline is RGB image.

As background prior techniques appear to have some limitations related to near-boundary-salient object, this work proposes an improved approach that refines the background dictionary instead of using it directly. This refinement provides substantial progress in saliency detection accuracy. Figure 1 shows the pipeline of the proposed method. The proposed approach utilizes the boundary conductivity term^8^, which suggests that an image region is more potential to be a portion of the background if it is strongly linked to the image border, especially for large and homogeneous backgrounds. By refining the background dictionary, regions close to the boundary and potentially be part of the salient object can be removed from the background dictionary; as a result, the salient object is identified more accurately. Once the refined background dictionary is obtained, the proposed method calculates the saliency values of the image regions by evaluating the reconstruction error of the regions using the refined background dictionary through sparse and dense representation^9,10^. Where a significant difference exists between a salient object region and the background dictionary, a large reconstruction error will be obtained as the dictionary failed to reconstruct that region, implying that the region has a large saliency value. Conversely, if an image region is similar to the background, a small reconstruction error will be obtained, indicating that the region acquires a small saliency value. So, the proposed approach can effectively handle the near-boundary salient object detection issue, a common issue in background prior-based techniques.

Also, this study focuses on improving salient object segmentation and preserving the object boundary using a pre-trained CascadePSP network^11^. This network overcomes the shortage of segmentation refinement approaches, such as graphical models (Conditional Random Field^12,13^) or region growing^14,15^, which are sensitive to initial seeds and typically rely on low-level features; they don't achieve significant improvement for refining the background prior-based saliency detection. The CascadePSP method refines an initial mask generated using the background prior-based method as input, which is a rough location of the object, in a cascading manner. It starts with a coarse initial mask and gradually refines the object's details by feeding the outputs from early levels into the later levels of the network. The Pyramid Scene Parsing Network (PSPNet)^16^ is used for pyramid pooling in the CascadePSP network, which is effective for capturing contextual information regardless of input resolution. As a result, the CascadePSP method generates a more refined and precise segmentation mask.

The principal contributions of this work can be summarized as follows: A segmentation refinement model, The CascadePSP^11^, is integrated with a background-prior-based saliency detection method to refine the saliency masks and segment salient objects. Where saliency masks are generated by dense and sparse reconstruction in terms of a refined background dictionary^10^, The outcomes provide compelling evidence for the significance of a refined background dictionary in saliency detection and demonstrate how CascadePSP boosts performance.

## Related work

In this section, we review related works in 2 categories^17–19^, including: (1) Traditional Saliency Detection models, (2) Deep-Learning Saliency Detection.

### Traditional saliency detection

Saliency detection methods can be broadly categorized into bottom-up and top-down. They have different characteristics and strengths, which can sometimes be complementary. Bottom-up approaches^20,21^ rely on low-level features such as intensity, color, and edges, making them robust and computationally efficient. On the other hand, top-down approaches^22,23^ utilize high-level features such as objectness, semantic information, and task-driven objectives, offering flexibility and adaptability. Some researchers^24,25^ aim to enhance saliency detection by developing integrated and hybrid approaches that combine the strengths of both bottom-up and top-down methods.

Local contrast methods estimate saliency values based on the nearest neighborhoods surrounding a region. Liu et al.^26^ use a conditional random field to combine local features, including multiscale contrasts and center-surround histograms. The method^27^ calculates pixel-level contrasts as local color dissimilarities with Gaussian distance weight. While these methods often highlight salient object boundaries rather than the entire object, they may respond to small, prominent regions in the background^28^. Limitations related to image noise, scene complexity, and robustness have led to exploring techniques such as boundary cues, global contrast measures, and adaptive thresholding^29^.

Global contrast approaches consider the entire image to estimate the contrast of its regions, providing a more reliable outcome than local models. Fang et al.^30^ represent patch features using the quaternion Fourier transform's amplitude spectrum. Despite their reliability, global contrast models may struggle with large salient objects or cluttered backgrounds. Various techniques, such as manifold ranking^31^ and deep learning approaches^32^, have been proposed to integrate local and global cues^33^ for refining salient regions.

Efforts to improve saliency detection involve incorporating local/global saliency detection with weighted-color channels, wavelet transform, feature learning, or PCA is explored. In^34^, an analysis of eye-tracking data is used to propose the RNCw model, which employs a channel-weighted color distance metric and spatial weighting to enhance region contrast. In^35^, Hierarchical Principal Component Analysis is applied to image layers generated through bit surface stratification, selecting the optimal saliency map based on information entropy. This approach might encounter difficulty in capturing complete object information when background objects share similar brightness levels and resolution. Lad et al.^36^ introduce an approach integrating global and local saliency detection through wavelet transform and learning-based saliency detection with a guided filter, but it is sensitive to parameter configurations. Wang et al.^37^ formulate saliency detection as a mathematical programming problem to learn a nonlinear feature mapping for multi-view features.

Several techniques based on background prior^38–40^ have been developed to address the limitations of contrast-based approaches, where using the background prior rather than the conventional local and global methods offers the incorporation of contextual background information. Background prior methods are built on the observation that the areas of an image closer to the image border are more probable to belong to the background. So, these methods use the image boundary areas as a template for the background and calculate the saliency value of the image regions as its feature contrast to the background template^41^. Wei et al.^42^ defined saliency as the feature distance to the image boundary along the shortest possible path. Meanwhile, Zhu et al.^8^ introduce a technique that measures saliency by analyzing the region's connectivity with the image border and its spatial location. The approach proposed in^43^ involves using manifold ranking on a multi-layer graph, considering both feature similarity and spatial proximity of superpixels. The method^43^ employs a two-step manifold ranking algorithm to calculate the saliency of each superpixel, incorporating background and foreground priors. Background prior framework has been effectively applied in numerous existing works^44,45^. These background-prior-based methods can effectively enhance salient object detection and suppress the background regions, even in cluttered scenes. However, their effectiveness strongly depends on the selection of background regions (dictionary). Challenges may arise in accurately identifying salient objects near the image boundary due to assumptions associated with the background prior or when the boundary is too flat to represent a cluttered background.

### Deep-learning saliency detection

The introduction of Fully Convolutional Neural Networks (FCN) in saliency map detection^46^ and semantic segmentation^47,48^ was a significant advancement at the time. Local features provided by FCN are insufficient for pixel labeling tasks, so a wide field-of-view contextual information is essentially integrated with local features. Many saliency detection models exploiting contextual information include image pyramid methods as multiscale inputs^49^ or dilated convolutions with different rates^50^.

Saliency detection methods^51–53^ often utilize encoder-decoder models. These models reduce the spatial dimensionality in the encoder stage to extract high-level information and then use a decoder to restore the spatial structure. Skip connections are also commonly used^53^ to obtain finer boundaries. Most Saliency detection models combine bilinearly up-sampled outputs at different strides (scales), leading to inaccurate labeling.

Song et al.^54^ introduced an innovative fusion framework incorporating a self-attention mechanism and a three-dimensional Gaussian convolution kernel to integrate background and multiscale frequency-domain features in salient detection. The approach proposed by Zhang et al.^55^ involves training a Rank-SVM classifier using object-level proposals and features from a region-based convolutional neural network (R-CNN). The saliency map for each image is then generated through a weighted fusion of its top-ranked proposals. It's important to note that the method's effectiveness depends on the quality of object proposals and R-CNN features, which may not be optimal for particular images.

Wang et al.^19^ comprehensively analyzed the evolution of salient object detection methods in the deep learning era. They delve into various deep learning architectures, including a Bottom-up/top-down network that refines rough saliency maps in the feed-forward pass by progressively incorporating spatial-detail-rich features from lower layers. Wang et al.^56^ proposed a method for salient object detection that merges multi-level pyramid attention mechanisms with salient edges to capture hierarchical features at varying scales and improve object boundary localization. Concurrently^57^, presented a model that infers salient objects from human fixations. This method involves the integration of human gaze data with deep learning techniques to predict saliency maps that closely align with human visual perception. Furthermore, Wang et al.^58^ proposed an iterative top-down and bottom-up inference network, demonstrating enhanced performance.

While deep-based methods have shown promising results in segmentation tasks, they often do not generate high-quality segmentations. These models face many challenges. One of the primary issues in salient object detection is handling complex scenes. This can make it difficult for CNN-based methods to detect the salient object accurately. Also, Salient objects can vary significantly in appearance, size, and shape, making it challenging to develop a universal saliency model that works well across all images and scenarios. Moreover, many CNN-based methods may not generalize well to new or unseen images, particularly if the images differ significantly from the training data.

On another side, some methods have complex network architectures with huge parameters, which require significant computational resources and memory. Several proposed methods require careful tuning of hyperparameters or may be sensitive to changes in the training data, which could impact their generalization performance. Also, CNNs can be difficult to interpret, making it challenging to understand why certain portions of the image are recognized as salient.

Therefore, researchers have explored several approaches to improve the segmentation quality and refine the results. Some approaches used graphical models such as Conditional Random Field^12,13^ or region growing^14,15^. These methods typically rely on low-level color features and do not adequately utilize high-level semantic context. Computational cost and memory constrain propagation-based approaches^59^ to refine the results of high-resolution images.

In an effort to enhance the performance of deep learning, Qin et al.^60^ introduced BASNet, a boundary-aware salient object detection method, integrating the refinement module into the prediction module to capture detailed boundary information. Similarly, Zhao et al.^61^ contributed EGNet, an edge guidance network that effectively integrates edge information into the saliency prediction process. Particular layers are trained explicitly to obtain the edge map, which is subsequently fused with the remaining layers to facilitate saliency detection. Moreover, Wu et al.^62^ proposed a partial decoder network with cascaded stages, each refining the saliency map progressively. Finally, Qin et al.^63^ presented U2-Net, a network that employs a nested U-structure equipped with skip connections, thereby enabling deeper and more robust saliency predictions.

Recently, there has been an increasing focus on developing separate refinement modules^64,65^ that can be holistically trained, enabling an end-to-end learning approach. They are typically used as the enhancement step after obtaining an initial segmentation, and their goal is to refine segmented objects. One challenge associated with refinement modules is that larger networks^64^ are more susceptible to overfitting, leading to poor generalization performance. On the other hand, shallow refinement networks^65^ have limited capacity for improving the accuracy of boundaries.

The structure of this paper is as follows. Section "Methods" describes the proposed method for detecting saliency maps using a refined background dictionary and the saliency mask refinement. Section "Experimental results and discussion" provides details on the experimental results and discussion obtained, while Section "Conclusions" presents the conclusions drawn from this study.

## Methods

The flowchart for the proposed system is shown in Fig. 1. The first step involves segmenting the image into visually cohesive regions and extracting the regional features from each region. In the second step, a refined background dictionary is generated by utilizing a measure that identifies the probability of the boundary regions being part of the background or the salient object. The third step involves estimating the saliency value independently for each region by computing the reconstruction errors of sparse and dense representations of the image regions using the refined background dictionary, which generates a saliency map. The saliency map is thresholded in the fourth step to produce an initial mask. Finally, the initial saliency mask is refined using the CascadePSP network to achieve an accurate and robust saliency image.

### Pixel-level image segmentation

In order to enhance the accuracy of saliency detection and increase processing efficiency, this study employs the Simple Linear Iterative Clustering (SLIC) algorithm^66^ for image segmentation and abstraction. This algorithm decomposes images into visually uniform regions known as superpixels that preserve edges. This approach achieves more robust saliency detection outcomes than saliency detection at the pixel level.

This study uses color as a visual feature to describe superpixels, given their crucial role in determining saliency. Specifically, the color descriptor of each superpixel is obtained as the mean value of its RGB and CIE-Lab color space. This feature effectively eliminates slight noise within homogeneous regions. Since SLIC generates superpixels with fairly regular shapes, the centroid $(x,y)$, which refers to the spatial location and average position of all pixels that belong to the superpixel, is used as an additional feature.

Therefore, the feature vector $f=[R,G,B,L,a,b,x,y]$ describes each superpixel. Subsequently, the image can be expressed as $F=[{f}_{1},{f}_{2},\dots ,{f}_{k}]$, where $k$ denotes the superpixels number. The initial background dictionary is denoted as $B=[{b}_{1},{b}_{2},\dots ,{b}_{k\mathrm{^{\prime}}}]$, where ${k}^{\mathrm{^{\prime}}}<< k$ signifies the number of superpixels present on the image boundary.

### Background dictionary refinement

This process aims to remove superpixels that are part of the salient object and are in contact with the image border from the background dictionary. As a result, the refined background dictionary only includes superpixels located on the image border that are not a part of the salient object. Zhu et al.^8^ introduced the concept of "background conductivity (BC)" for superpixels as the degree to which a superpixel is related and belongs to the image boundary. Thus, if a superpixel is strongly linked to the image's border (gives a significant BC value), it is more likely to be a portion of the background. In contrast, a salient object is typically less associated with the image border, even if it is near it.

The object's boundary conductivity is the ratio of its perimeter along the boundary of the image to its area. The area's square root is used to achieve scale invariance, ensuring that the boundary conductivity remains consistent over various image patch scales. As a result, the formula for an object's boundary conductivity ($$BC)$$ is as follows:

1 $$BC\left(p\right)= \frac{ Length \left(p\right) on\, image\, boundary}{\sqrt{Area\left(p\right)}}$$

As a result, estimating the boundary conductivity for a superpixel requires calculating the length along the image boundary and the area of the homogeneous region that the superpixel relates to. The area is defined by the contribution of other superpixels to a specific one. If two superpixels are highly similar (i.e., located in a homogeneous area), one superpixel introduces a unit area to another. The formula for calculating the area associated with a superpixel *p* is as follows:

2 $$Area\left(p\right)= \sum_{i=1}^{k}{\text{exp}}(- \frac{{d}_{geo}^{2}(p,{p}_{i})}{2{\sigma }^{2}})= \sum_{i=1}^{k}A(p,{p}_{i}),$$

3 $${d}_{geo}\left(p,q\right)= \genfrac{}{}{0pt}{}{min}{p={p}_{1},{p}_{2},\dots , {p}_{n}=q}\sum_{j=1}^{n-1}{d}_{app}\left({p}_{j},{p}_{j+1}\right),$$

where ${d}_{app}\left(p,q\right)$ is a metric that quantifies the Euclidean distance between the pair feature vectors of superpixels $\left(p,q\right).$ Eq. (3) formulates a method for calculating the geodesic distance, ${d}_{geo}\left(p,q\right)$ between a pair of superpixels $\left(p,q\right)$, computed as the total weight of edges along the shortest route connecting them, with minimum cost. According to Eq. (2), if two superpixels are located in a homogeneous region, ${d}_{geo}\left(p,q\right)$ will be very small and tends to be zero and consequently $(A(p, q) \approx 1)$. As a result, two superpixels contribute an area unit to each other. Experimental results^8^ show that $\sigma = 10$. Similarly, the length associated with a superpixel $p$ is calculated using Eq. (4) that measures the contribution of image boundary's superpixels to superpixel $p$, where $\delta =1$ for superpixels located on the image boundary and zero otherwise, and dot '.' represent multiplication operation.

4 $$length\left(p\right)= \sum_{i=1}^{N}A\left(p,{p}_{i}\right).\delta \left({p}_{i} \in imageboundary\right)$$

Once the background conductivity has been normalized, a threshold is chosen to eliminate superpixels with low background conductivity from the background $B$, in order to achieve a more precise background ${B}^{*}$ . An adaptive threshold $(\tau )$ is estimated based on the input image using Eq. (5).

5 $$\tau ={BC}_{Bmax}-K var\left(B\right),$$

where ${BC}_{Bmax}$ refers to the maximum background conductivity, and $var(B)$ refers to the variance of the unrefined (initial) background dictionary $B$. The value of parameter $K$ is experimentally set to 4.

### Saliency measurement estimation

This study approaches saliency detection in images by estimating the reconstruction error of the superpixels using the background dictionary. It is assumed that a significant difference exists between the reconstruction errors of background and foreground superpixels when utilizing the same dictionary for representation. The feasibility of this approach rests upon the use of the optimal background dictionary. This leads to accurately identifying the salient regions by comparing the reconstruction errors of the background and foreground superpixels.

Two representations of the superpixel, as represented by a D-dimensional feature vector, are utilized to determine the significance of each superpixel, including dense and sparse representations. Dense appearance models provide a more general and comprehensive representation of the background dictionary, while sparse models create distinct and concise representations. However, dense appearance models are known to be more susceptible to noise and may not be as effective in identifying salient objects in cluttered scenes through reconstruction errors. Conversely, sparse representation solutions are less consistent; sparse coefficients may differ among similar regions, leading to inconsistent saliency detection outcomes. This study uses complementary dense and sparse representations to model superpixels and assess their significance through the reconstruction error.

**Figure 2:**
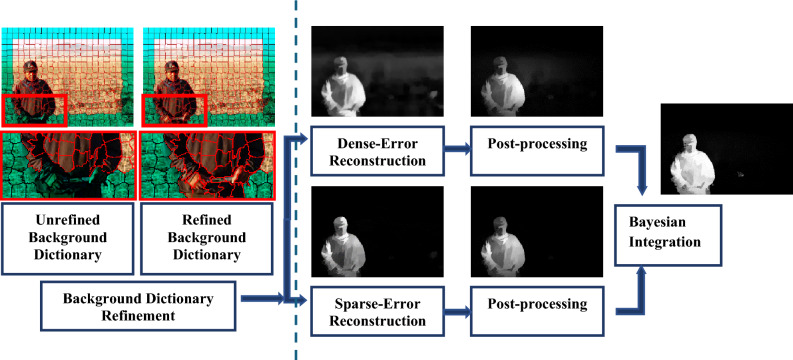
Flowchart of the proposed saliency detection method. Left: visualization for background dictionary refinement (section "Background dictionary refinement"), Right: Visualization for Saliency Measurement Estimation (section "Saliency measurement estimation").

The process of computing saliency measures using the reconstruction errors of dense and sparse representation is illustrated in Fig. 2. The first step involves reconstructing all image superpixels utilizing the refined background dictionary. Once the reconstruction errors obtained have been normalized to the range of [0, 1], a propagation method is introduced to leverage the advantage of local contexts and enhance the outcomes. Finally, pixel-level saliency is determined by considering the reconstruction errors at multiscale superpixels.

#### Dense reconstruction

A superpixel is more probable to be classified as a segment of the foreground if its reconstruction error is greater than that similar to the background dictionary atoms. To compute the reconstruction error of each superpixel, A dense appearance model is created by implementing Principal Component Analysis (PCA) on the refined background dictionary. The PCA bases are formed by using the eigenvectors ${(V}_{{B}^{*}})$ associated with the largest eigenvalues that are extracted from the covariance matrix of the refined background dictionary ${(B}^{*})$. This enables the computation of the reconstruction coefficient $\left({\gamma }_{i}\right)$ for superpixel $i$ by:

6 $${\gamma }_{i}={{V}_{{B}^{*}}}^{T} \left({f}_{i}- \overline{f }\right),$$

where $\left({f}_{i}\right)$ is the feature descriptor of superpixel $i$ , $\overline{f }$ is the mean feature descriptor of all superpixels $F$. Then, dense reconstruction error can be calculated as:

7 $${{\epsilon }^{D}}_{i}= {\Vert {f}_{i}-\left({V}_{{B}^{*}} {\gamma }_{i}+\overline{f }\right)\Vert }_{2}^{2}.$$

These normalized reconstruction errors, which typically fall within the range of [0, 1], are directly proportional to the saliency measures.

**Figure 3:**
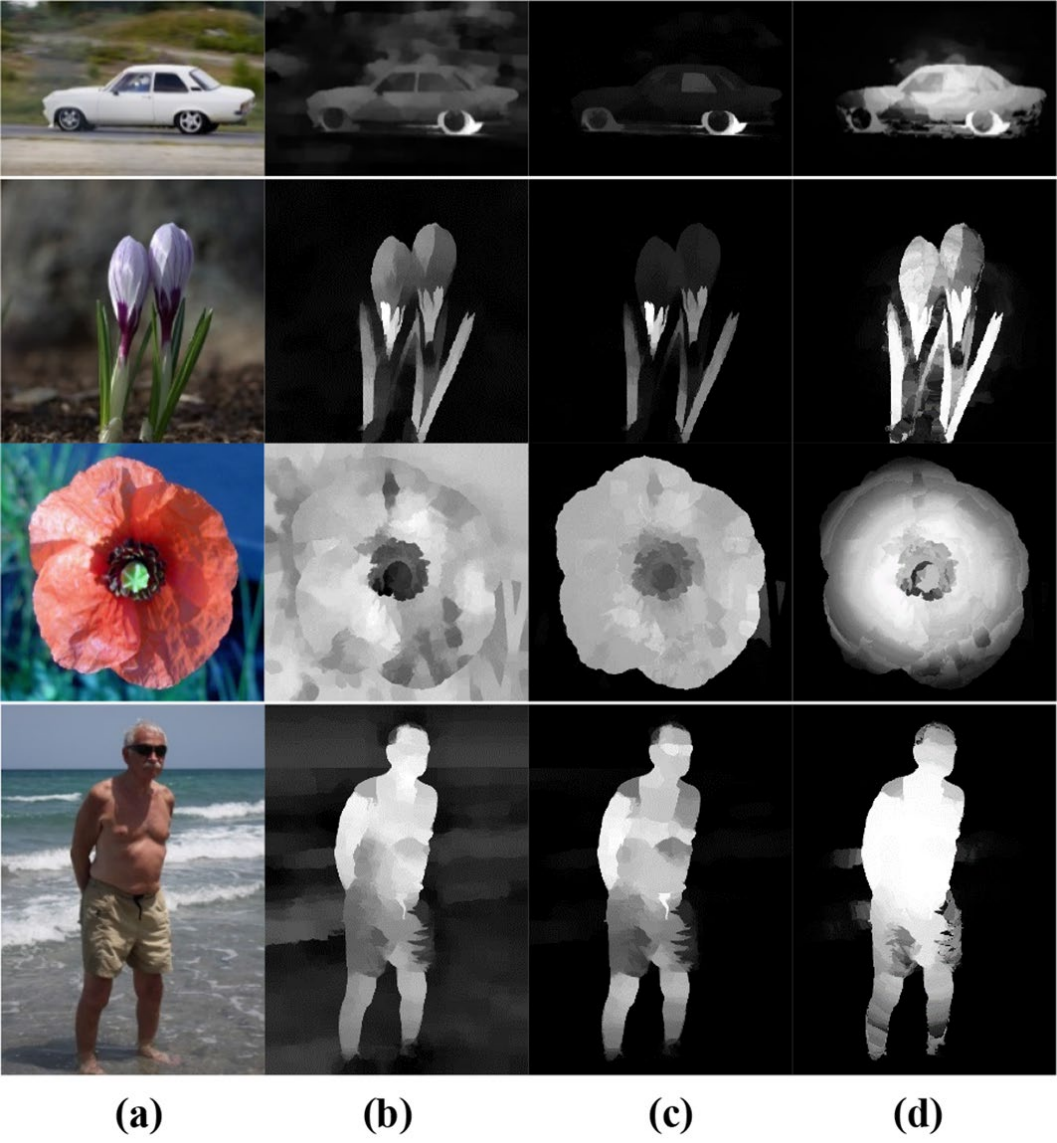
(**a**) RGB image, (**b**) Dense Representation-Saliency Map, (**c**) Sparse Representation-Saliency map, (**d**) Integrated saliency map.

In dense representations, data points are modeled as a multivariate Gaussian distribution in feature space. This approach can pose a challenge when attempting to capture multiple scattered patterns. Figure 3a,b shows an instance where some background superpixels exhibit large reconstruction errors. This can lead to imprecise saliency measures. On the other side, this representation successfully highlights the salient object despite the background suppression problems.

#### Sparse representation

Sparse reconstruction of each superpixel is achieved by utilizing all superpixels in the refined background dictionary as the bases to encode the superpixel $i$ and calculate the sparse reconstruction error ${({\epsilon }^{S}}_{i})$ as:

8 $${{\epsilon }^{S}}_{i}= {\Vert {f}_{i}-{B}^{*} {\alpha }_{i}\Vert }_{2}^{2}.$$

where, the sparse representation coefficient ${\alpha }_{i}$ is given by:

9 $$\begin{array}{c}min\\ {\alpha }_{i}\end{array}{\Vert {f}_{i}- {B}^{*} {\alpha }_{i}\Vert }_{2}^{2}+ \lambda {\Vert {\alpha }_{i}\Vert }_{1}$$

As all superpixels in the refined background dictionary are considered as bases functions, sparse reconstruction errors are highly effective in suppressing the background, particularly in cluttered images, as demonstrated in Fig. 3c**.** It is noteworthy that sparse reconstruction errors are more robust in dealing with complex backgrounds. Therefore, dense, and sparse representations complement each other in measuring saliency.

**Figure 4:**
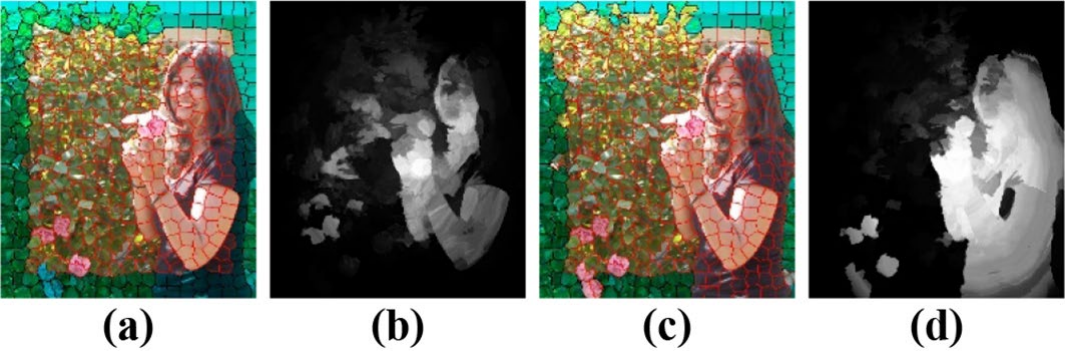
(**b**) Saliency map obtained using (**a**) the initial background dictionary (Green). (**d**) The proposed saliency map using the refined background dictionary (**c**).

The choice of the bases of the background dictionary has a substantial great influence on the resulting saliency values as they affect the reconstruction errors. Figure 4c,d shows the impact of using the refined background dictionary over the initial background dictionary Fig. 4a,b. A more reliable background dictionary that excludes the salient object's segments on the image boundary can enhance saliency outcomes.

#### Saliency maps post-processing and integration

This study presents a method for enhancing the accuracy of dense and sparse appearance models by smoothing reconstruction errors using context-aware error propagation. The method involves clustering $k$ image super-pixels into $N$ clusters via the K-means clustering method based on their D-dimensional feature vectors. The superpixels are sorted according to their reconstruction errors and sequentially processed within each cluster. The propagated error of a superpixel in cluster $N$ is adjusted by considering its context, which includes other superpixels in the same cluster.

Two factors are considered when estimating the propagated reconstruction error: the weighted average of the reconstruction errors of all members in the same cluster and the initial reconstruction error. Considering a superpixel's appearance-based local context, its reconstruction error can be more accurately estimated. The weight assigned to each superpixel's context is determined by a Gaussian distribution that normalizes the similarity between the superpixel and other members of its cluster.

The reconstruction errors at multiple scales are integrated and refined using an object-biased Gaussian function to create a full-resolution saliency map. This allows for assigning saliency values to individual pixels instead of individual superpixels. To address the scale issue, the pixel-level reconstruction error is computed through the weighted mean of the multiscale propagated reconstruction errors; this weight is determined by the similarity between the pixel and its corresponding superpixel^9^.

Previous research has indicated that certain saliency detection datasets exhibit a center bias^17^. To account for this, recent approaches have incorporated a center prior in the form of a 2D-Gaussian model with the mean set to the image center's coordinates^67^. However, this approach is not always effective, as the center of an image may not necessarily include the salient objects. Instead, an object-biased 2D-Gaussian distribution that uses the object center derived from pixels error as the mean of the Gaussian distribution is employed^9^.

The saliency values obtained from dense and sparse reconstruction errors complement each other. Bayesian inference is used to integrate these two measures effectively by allowing the two maps to serve as priors to each other to highlight salient objects uniformly (Fig. 3d).

### Saliency mask initialization

**Figure 5:**
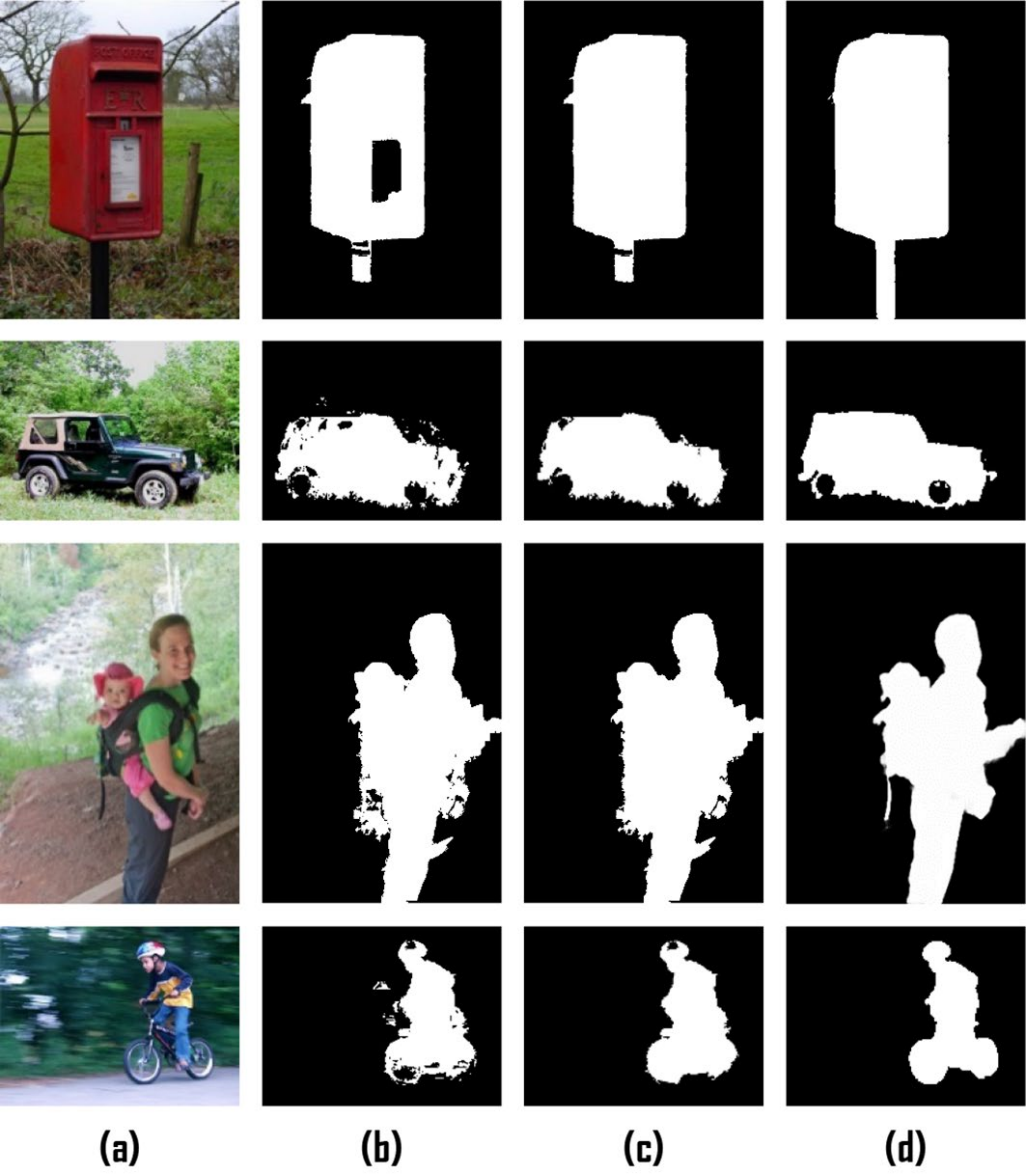
(**a**) Original Image. (**b**) Threshold-Saliency Map. (**c**) Initial Mask of refinement stage. (**d**) Refined Mask.

To transform the saliency map into a more unified salient object detection segmentation (Fig. 5a–d), the saliency map is thresholded to produce an image mask (Fig. 5b). Then, this Mask is processed to remove very small objects that are about of size less than 10% of the maximum object size. After that, simple morphological operations are used to fill in the small holes. Finally, the generated Mask is used as the initial Mask (Fig. 5c) for the segmentation refinement stage to produce the refined Mask (Fig. 5d).

### Saliency mask refinement

The CascadePSP refinement network proposed in^11^ is used to refine the initial Mask generated from saliency map detection. The CascadePSP approach begins with the initial Mask, which roughly identifies the object's location. The structure of CascadePSP is designed to generate a series of progressively refined segmentation masks, starting from this initial, coarse Mask. The network first predicts the general structure of the object using the early levels' coarse outputs. These outputs are then used as inputs to the network's later levels, allowing further refinement of the object's details.

**Figure 6:**
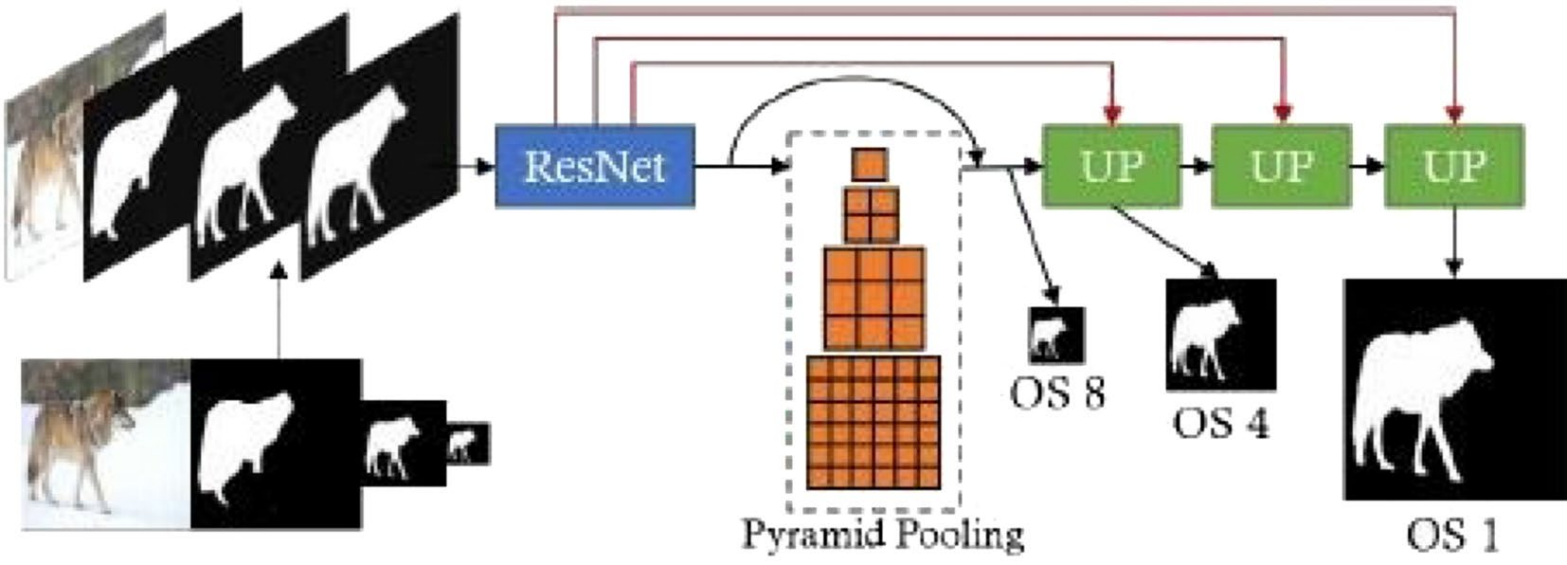
Single Refinement module structure refines segmentation by taking inputs from three levels of segmentation with different output strides. Skip-connections are denoted by red lines^11^.

This network is based on a single refinement module (RM) that can be used in cascade form to achieve global and local refinements. The single refinement module (RM)^11^ shown in Fig. 6 uses an image with several incomplete segmentation masks at various scales as input. Using multiscale inputs to refine the segmentation allows the network to fuse the mask features from various scales and collect boundary and structure details. Therefore, the lower-resolution masks are bilinearly upscaled and concatenated with the RGB image used as the network's input.

The stride 8-output feature vector is extracted from the input by PSPNet^16^ with ResNet-50^68^ as the backbone. Many errors related to contextual relationships and global information for different receptive fields motivate the author to use a suitable global-scene-level-prior to improve performance. So, the pyramid pooling module of sizes of (1, 2, 3, and 6), as in^16^, is used to tackle this issue. The model generates stride 1, 4, and 8 outputs covering finer and coarser structures.

It would be necessary to pass information across the network to tackle the problem of losing image details at deeper layers. As a result, skip connections are used to connect the backbone and the up-sampling block. The skip-connected features and the bilinearly up-sampled main branch features are concatenated and then processed by two ResNet blocks. A two-layer 1 × 1 convolution followed by a sigmoid function is used to obtain the segmentation output^11^.

Multiple loss functions are used to obtain optimal outcomes^11^. Since the coarse refinement ignores local features and concentrates on the global structure, the cross-entropy loss is applied to the coarse output (stride 8). L1 + L2 loss is used for the finer output (stride 1), where the finest refinement seeks to use local features to achieve pixel-wise quality. For intermediate output (stride 4), the average of L1 + L2 loss and cross-entropy loss is used. Furthermore, L1 loss on the gradient of the finest output (stride 1) is applied to achieve more accurate boundary refinement. The gradient is easily estimated by a 3-kernel average filter followed by a Sobel operator^69^.

## Experimental results and discussion

### Datasets and evaluation measures

Three datasets are used to evaluate the proposed saliency map detection. They include the ASD dataset^70^, which is relatively simple and contains 1000 images. The other two datasets are more challenging. MASR10K^28^ includes 10,000 images of low contrast, single and multiple objects with a complex background that includes reflections, motions, and shadows. ECSSD^71^ is the most challenging dataset, which includes 1000 images of a complex scene with multiple (1–4) objects in a more complicated background.

Multiple metrics are used to assess the performance of the proposed framework^17–19^. Since the mean absolute error (MAE) score indicates the closeness of the saliency map to ground truth, it is a useful tool for evaluating object segmentation tasks. MAE is considered the average per-pixel error and is given by:

10 $$MAE= \frac{1}{W\times H}\sum_{i=1}^{H}\sum_{j=1}^{W}\left|S\left(i,j\right)-G(i,j)\right|,$$

where $W$ and $H$ represent the width and height, respectively, of ground truth $G$ and saliency map $S$.

Moreover, The ROC curve is a graph that displays how well a classification model performs across all classification thresholds. Therefore, the (AUC) area in two dimensions beneath the complete ROC curve is calculated to quantitative this measure.

In the same context, The Precision-Recall (PR) curve for the entire dataset is developed by averaging the PR curves over images containing the dataset. The PR curve was employed to assess the similarity between the binary masks generated from the saliency map (at various threshold levels within the range of T ∈ [0, 255]) and the ground truth. The F1-Measure was utilized as a harmonic mean of these two performance indicators to integrate precision and recall into a single metric. F1-Measure is given by:

11 $${\text{F}}1-{\text{Measure}}=\frac{2*{\text{Precision}}*{\text{Recall}}}{{\text{Precision}}+{\text{Recall}}+{\text{eps}}} .$$

Also, to emphasize precision over recall, Fβ-Measure^72^ is used as that is the weighted harmonic mean for precision and recall and given by:

12 $$\mathrm{F\beta }-\mathrm{Measure }= \frac{\left(1 + {\beta }^{2}\right)*{\text{Precision}}*{\text{Recall}}}{\left({\beta }^{2}*{\text{Precision}}+{\text{Recall}}\right)} ,$$

where ${\beta }^{2}=0.3$, as recommended in^28^.

As the S-measure^73^ considers both the region-based similarity $Sr$ and the object-aware structure similarity $So$ between the saliency map and the ground truth, it is comprehensively used to assess the accuracy and consistency of saliency maps. S-measure calculates as follows:

13 $$S-measure = \propto \cdot So + \left(1 - \propto \right)\cdot Sr,$$

where $\propto$ set to 0.5 as in^73^.

The E-measure^74^, also known as the Enhanced-alignment measure, is a metric that combines global image-level statistics and local pixel-matching information. So, E-measure is used to evaluate saliency detection performance comprehensively.

### Comparison with traditional saliency detection methods

#### Proposed saliency detection (without mask refinement)

Quantitative comparison was conducted between the proposed Saliency map (without Mask Refinement) and the traditional seventeen state-of-the-art techniques, including FES^75^, GR^76^, MC^77^, SeR^78^, SIM^79^, SR^20^, SWD^80^, DSR^9^, SMD^81^, HLR^82^, Method^83^, SOD_TSWA^84^, RNCw^34^, Methods proposed in^35,36^, NFM^37^, and^43^. The visual results and some of the evaluation metrics are unavailable for some techniques. The performance of the proposed saliency detection is compared with at least nine competing methods in terms of MAE, F1-measure, Fβ-measure, S-measure, E-measure, and AUC, as illustrated in Tables 1, 2, and 3.

**Table 1:** Methods comparison on the ASD Dataset.

Method	MAE	F1-Measure	Fβ-measure	E-measure	S-Measure	AUC
**Traditional saliency detection**						
FES^75^	0.165	0.411	0.684	0.833	0.633	0.928
GR^76^	0.161	0.520	0.848	0.887	0.806	0.977
MC^77^	0.093	0.648	0.895	**0.937**	0.858	0.980
SeR^78^	0.312	0.330	0.430	0.652	0.565	0.819
SIM^79^	0.402	0.263	0.199	0.421	0.483	0.790
SR^20^	0.241	0.275	0.459	0.731	0.543	0.794
SWD^80^	0.266	0.345	0.604	0.737	0.656	0.924
DSR^9^	0.080	0.715	0.847	0.916	0.854	0.979
^35^	–	–	–	–	–	0.924
**Proposed saliency method**						
Without mask refinement	0.074	0.763	0.851	0.916	0.861	**0.981**
Initial mask	0.059	0.806	0.823	0.905	0.848	0.915
Refined mask	**0.045**	**0.851**	**0.866**	0.924	**0.885**	0.936
**Deep-learning saliency method**						
BASNet^60^	0.033	0.902	0.903	0.951	0.924	–
EGNet^61^	0.032	0.906	0.898	0.955	0.926	–
CPD^62^	0.033	0.902	0.896	0.952	0.923	–
U2NET^63^	0.030	0.909	0.909	0.955	0.931	–

The best performance among the traditional saliency detection by **bold**.

**Table 2:** Methods Comparison on the ECSSD Dataset.

Method	MAE	F1-Measure	Fβ-measure	E-measure	S-Measure	AUC
**Traditional saliency detection**						
FES^75^	0.212	0.333	0.598	0.740	0.560	0.873
GR^76^	0.284	0.351	0.512	0.626	0.618	0.876
MC^77^	0.202	0.455	0.699	0.788	0.693	**0.926**
SeR^78^	0.404	0.274	0.246	0.482	0.458	0.690
SIM^79^	0.433	0.266	0.134	0.350	0.453	0.729
SR^20^	0.311	0.244	0.366	0.637	0.488	0.708
SWD^80^	0.318	0.327	0.499	0..632	0.598	0.871
DSR^9^	0.171	0.514	0.689	0.787	0.685	0.914
SMD^81^	0.227	–	0.517	–	–	0.775
HLR^82^	0.176	–	0.545	–	–	0.820
^83^	0.262	–	0.572	0.688	0.583	–
SOD_TSWA^84^	0.313	–	0.307	–	–	–
RNCw^34^	0.173	–	–	–	0.669	–
^35^	–	–	–	–	–	0.799
^36^	0.200	–	–	–	0.728	0.811
NFM^[ 37^	0.157	–	0.514	–	0.705	0.842
^43^	0.168	–	–	–	**0.763**	–
**Proposed saliency method**						
Without mask refinement	0.173	0.516	0.673	0.780	0.680	0.909
Initial mask	0.155	0.614	0.660	0.769	0.688	0.786
Refined mask	**0.131**	**0.667**	**0.715**	**0.797**	0.737	0.820
**Deep-learning saliency method**						
^54^	0.162	–	–	–	0.719	0.802
KSR^55^	0.134	0.640	0.644	0.771	0.733	0.824
BASNet^60^	0.037	0.879	0.904	0.921	0.916	–
EGNet^61^	0.037	0.92	0.903	0.927	0.925	–
CPD^62^	0.037	0.917	0.898	0.925	0.918	–
U2NET^63^	0.033	0.892	0.91	0.924	0.928	–
^85^	0.060	–	0.882	0.907	0.869	–
PerGAN^86^	0.052	–	0.878	–	–	–
GSCINet^87^	0.034	–	0.911	0.953	–	–

The best performance among the traditional saliency detection by **bold**.

**Table 3:** Methods Comparison on the MSRA10K Dataset.

Method	MAE	F1-Measure	Fβ-measure	E-measure	S-Measure	AUC
**Traditional saliency detection**						
FES^75^	0.185	0.388	0.687	0.805	0.600	0.908
GR^76^	0.198	0.485	0.745	0.789	0.744	0.955
MC^77^	0.145	0.576	**0.836**	0.879	0.785	0.955
SeR^78^	0.310	0.352	0.429	0.630	0.571	0.809
SIM^79^	0.388	0.293	0.229	0.431	0.507	0.800
SR^20^	0.249	0.296	0.490	0.720	0.546	0.805
SWD^80^	0.267	0.367	0.610	0.713	0.662	0.912
DSR^9^	0.121	0.656	0.807	0.870	0.781	0.954
SMD^81^	0.104	–	0.704	–	–	0.847
HLR^82^	0.104	–	0.705	–	0.847	0.854
SOD_TSWA^84^	0.279	–	0.324	–	–	–
^36^	0.098	–	–	–	0.841	0.879
NFM^37^	0.106	–	0.765	–	**0.848**	0.937
^43^	0.122	–	–	–	0.837	–
**Proposed saliency method**						
Without mask refinement	0.114	0.678	0.810	0.875	0.791	**0.958**
Initial mask	0.090	0.760	0.791	0.871	0.801	0.868
Refined mask	**0.074**	**0.801**	0.832	**0.889**	0.838	0.891
**Deep-learning saliency method**						
^54^	0.092	–	–	–	0.827	0.843
BASNet^60^	0.041	0.901	0.892	0.938	0.916	–
EGNet^61^	0.045	0.906	0.878	0.935	0.909	–
CPD^62^	0.045	0.894	0.878	0.934	0.907	–
U2NET^63^	0.041	0.901	0.892	0.938	0.916	–

The best performance among the traditional saliency detection by **bold**.

The proposed saliency detection method outperforms competing approaches in almost all measures, according to Table 1 on the ASD dataset. It achieves excellent results, ranking first in measures such as MAE, F1-measure, S-measure, and AUC, and second in terms of Fβ-measure and E-measure.

When comparing with competing approaches on the complex scenes dataset (ECSSD), the proposed saliency detection performs the best in terms of F1-measure and third best in Fβ-measure, E-measure, S-measure, and AUC, as shown in Table 2.

On MSRA10K (Table 3), the largest dataset, the proposed saliency detection achieves the highest F1-measure and AUC score performance. The proposed method ranks second among the competing approaches in the Fβ-measure and S-measure while achieving comparable results for other measures. These findings indicate that the proposed saliency detection method is among the top three techniques across the three datasets.

**Figure 7:**
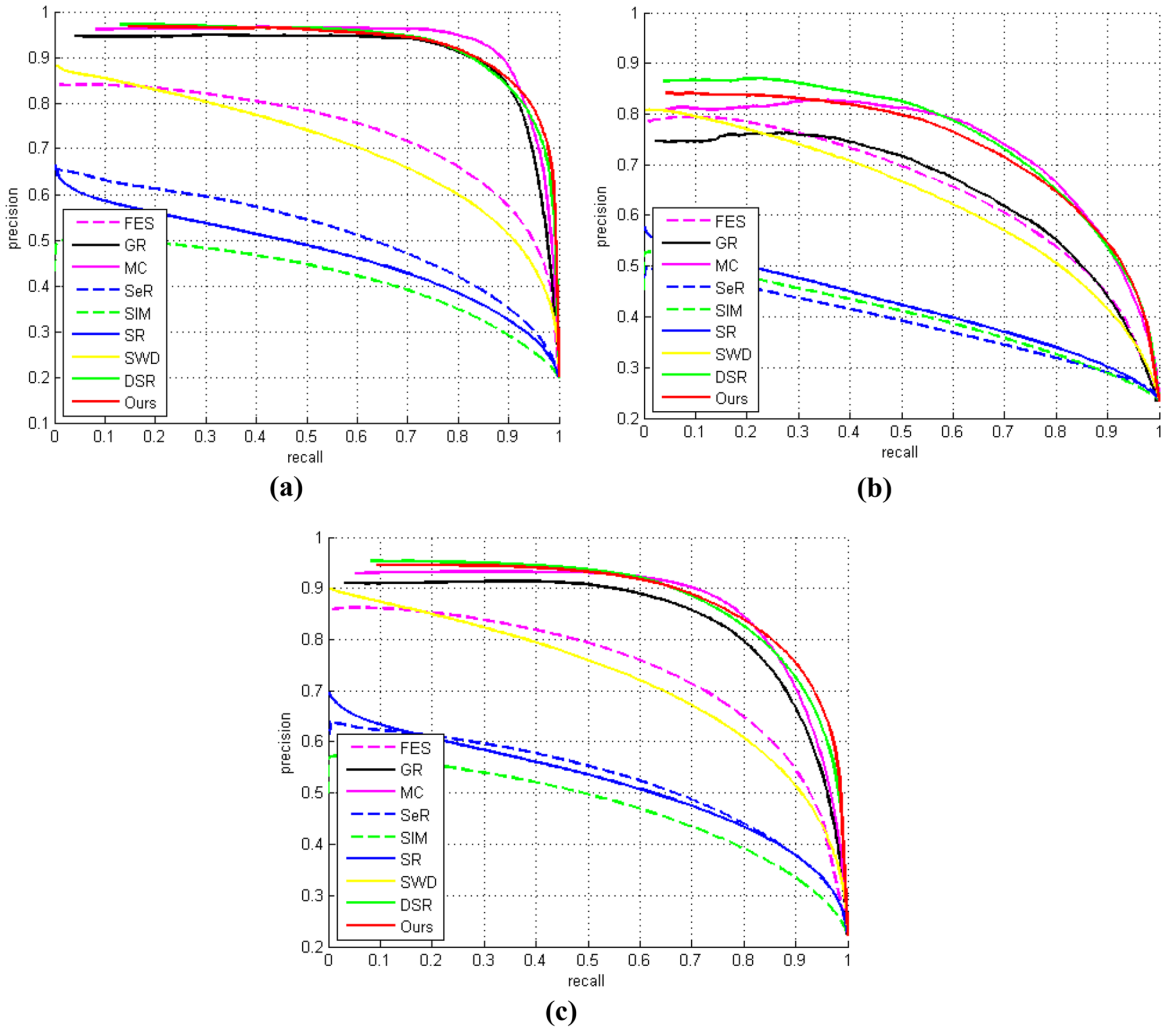
Precision-Recall curves for (**a**) ASD, (**b**) ECSSD, and (**c**) MSRA10K datasets.

Furthermore, the performance of the proposed saliency detection method was evaluated using the precision-recall (PR) curve, as depicted in Fig. 7a–c; the results indicate that the proposed method performed favorably and ranked among the three leading techniques.

**Figure 8:**
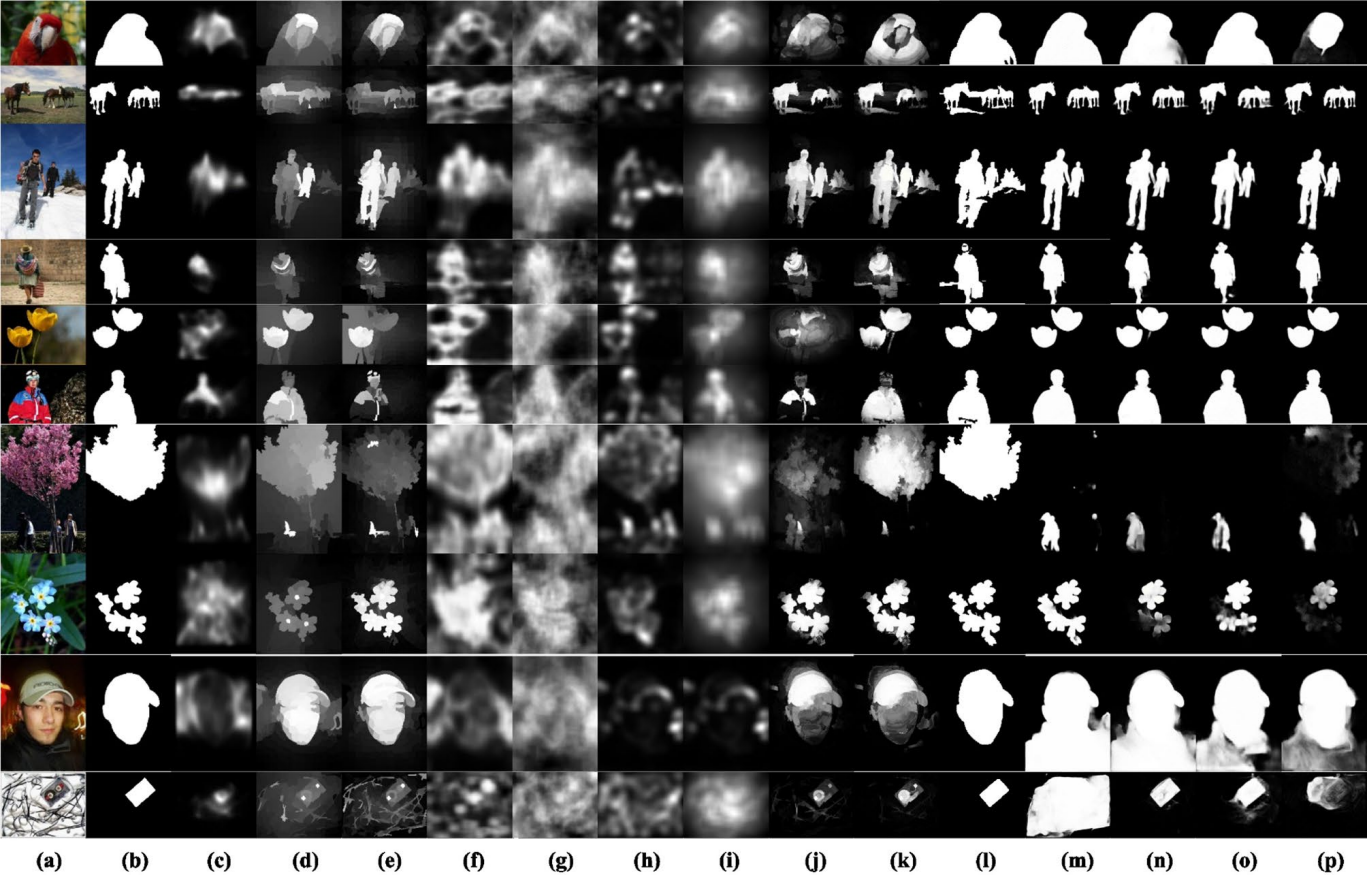
Visual comparison of the proposed Saliency maps against the state-of-the-art approaches. (**a**) Original image, (**b**) Ground truth, (**c**) FES^75^, (**d**) GR^76^, (**e**) MC^77^, (**f**) SeR^78^, (**g**) SIM^79^, (**h**) SR^20^, (**i**) SWD^80^, (**j**) DSR^9^, (**k**) The Proposed Saliency Method (Without Mask Refinement), (**l**) The Proposed Refined Mask, (**m**) BASNet^60^, (**n**) EGNet^61^, (**o**) CPD^62^, and (**p**) U2Net^63^.

Figure 8a–k presents visual comparisons between the saliency maps produced by the proposed method (Fig. 8k) and those generated by traditional state-of-the-art techniques (Fig. 8c–j). The proposed method demonstrates superior performance compared to the traditional techniques in detecting the near-boundary salient objects, where the increased ability to highlight the salient objects uniformly suppresses the background effectively and produces favorable visual outcomes for multiple objects with low contrast. Also, Fig. 8c–j illustrates that some traditional methods exhibit markedly inferior performance compared to the proposed method. In contrast, others are primarily designed for object localization, not for accurate detection.

#### Mask refinement

Tables 1, 2, and 3 give a fair comparison between two binary (initial and refined) Masks. In addition to MAE, F1-Measure, Fβ-measure, S-measure, E-measure, and AUC; The intersection-over-union (IOU) (Table 4) is used to evaluate the two Masks and how these masks are close to the ground truth. All evaluation measures demonstrate the preference for the refined Mask over the initial one and the original saliency map over the three datasets. As shown in Table 4, the refined Mask significantly improves IOU (at least 5%-IOU more than the initial Mask).

**Table 4:** Intersection-over-union (IOU) for Initial Mask versus Refined Mask.

	ASD	ECSSD	MSRA10K
Initial mask	0.767	0.541	0.699
Refined mask	0.822	0.607	0.754

Comparing the refined Mask to the initial Mask, the improvement margin is between 1.43% for MAE and 6.6% for IOU. On the other hand, the refined Mask improved the saliency map with a margin between 0.75% for the E-measure and 15.19% for the F1-measure. At the same time, the AUC of the refined Mask is less than that of the saliency map. This issue is because the saliency map has more gray levels than the refined Mask, which appears as a binary image. Compared to traditional methods (Tables 1, 2, and 3), these findings indicate that the refined Mask is among the top two techniques across the three datasets (The top one for the most challenging dataset, ECSSD, for almost measures). Tables 5 and 6 show the percentage of images in each dataset with MAE less than 10% and IOU greater than 90%, respectively.

**Table 5:** Percentage of images with IOU greater than a certain value.

IOU	ASD	ECSSD	MSRA10K
> 0.9	53.90%	14.51%	33.79%
> MEAN IOU	69.10%	52.45%	59.71%

**Table 6:** Percentage of images with MAE Smaller than a certain value.

MAE	ASD	ECSSD	MSRA10K
< 0.1	85.70%	50.05%	73.97%
< MEAN MAE	73.30%	60.16%	65.89%

**Figure 9:**
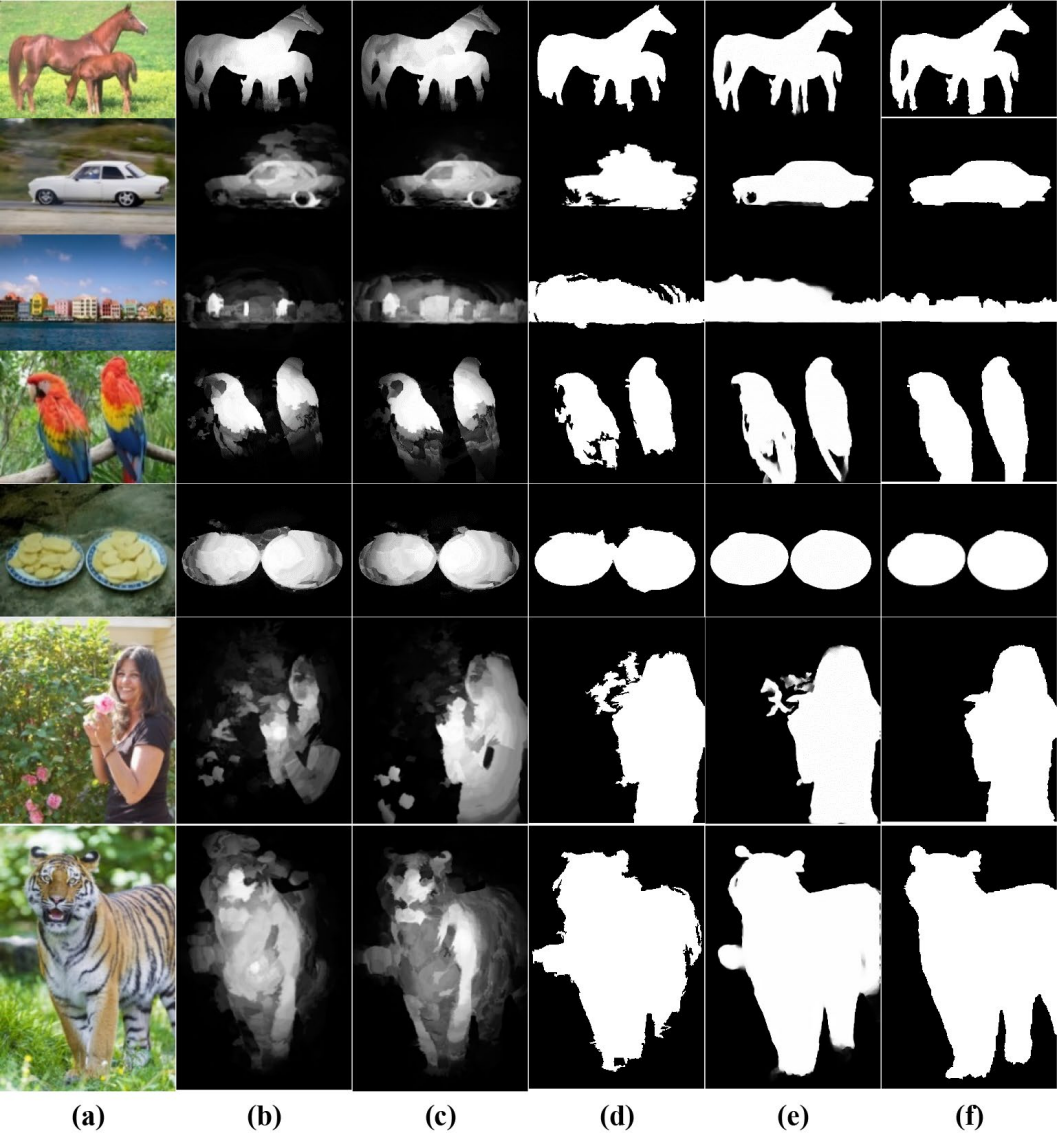
(**a**) Original image. (**b**) Saliency Map (using background dictionary directly without refining it). (**c**) Proposed Saliency Map (using refined background dictionary). (**d**) Initial Mask. (**e**) Refined Mask. (**f**) Ground truth.

Figure 9 demonstrates a visual preference for the refined Mask (Fig. 9e) over the saliency map (Fig. 9c) and the initial Mask (Fig. 9d) to segment the multiple salient objects, low object-background contrast, and near boundary objects. Also, Fig. 9c shows the advantage of refining the background dictionary over DSR^9^ using the background dictionary directly without refining it (Fig. 9b).

**Figure 10:**
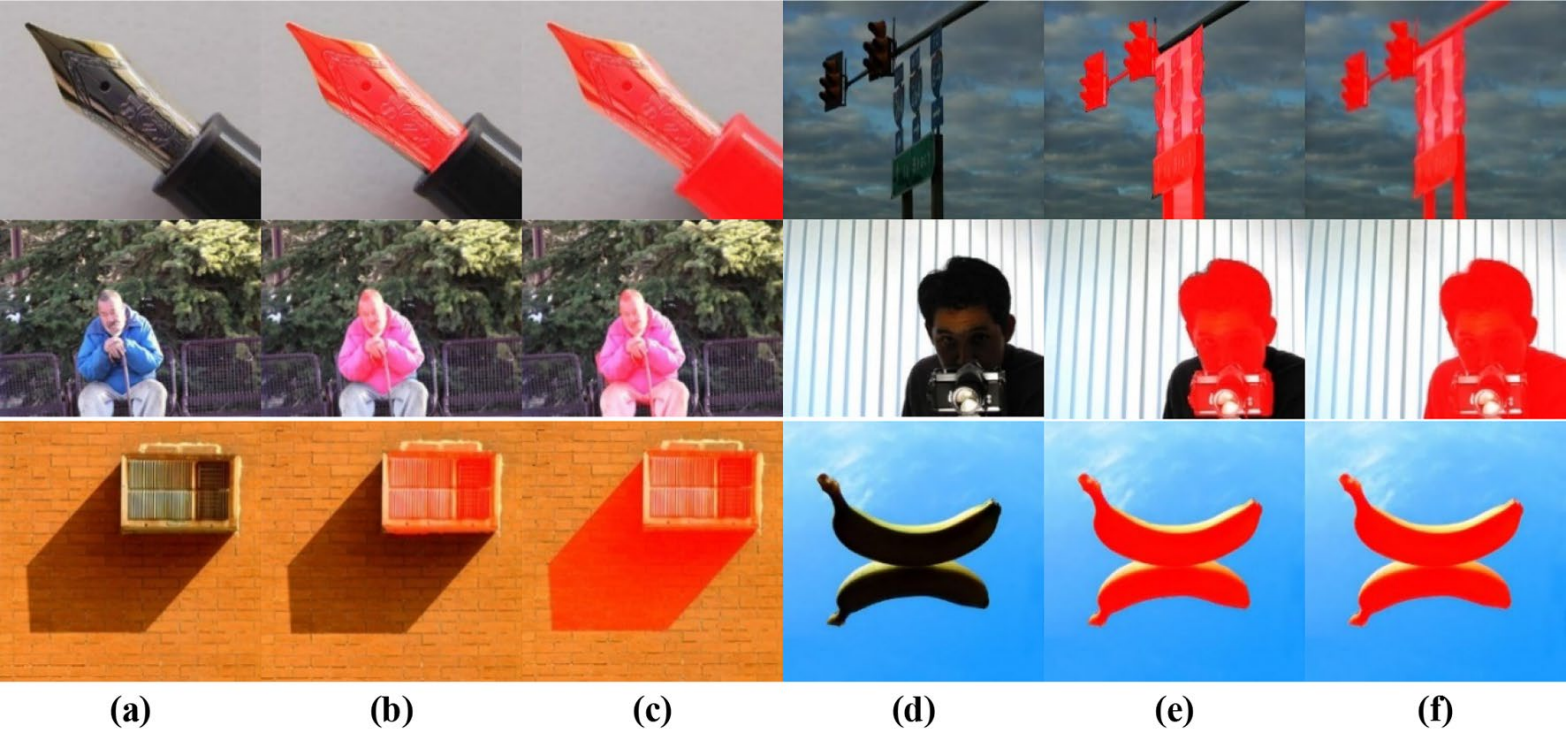
(**a**–**d**) Original Images. (**b**–**e**) highlighted salient object by Ground truth Mask. (**c**–**f**) highlighted salient object by refined Mask.

**Figure 11:**
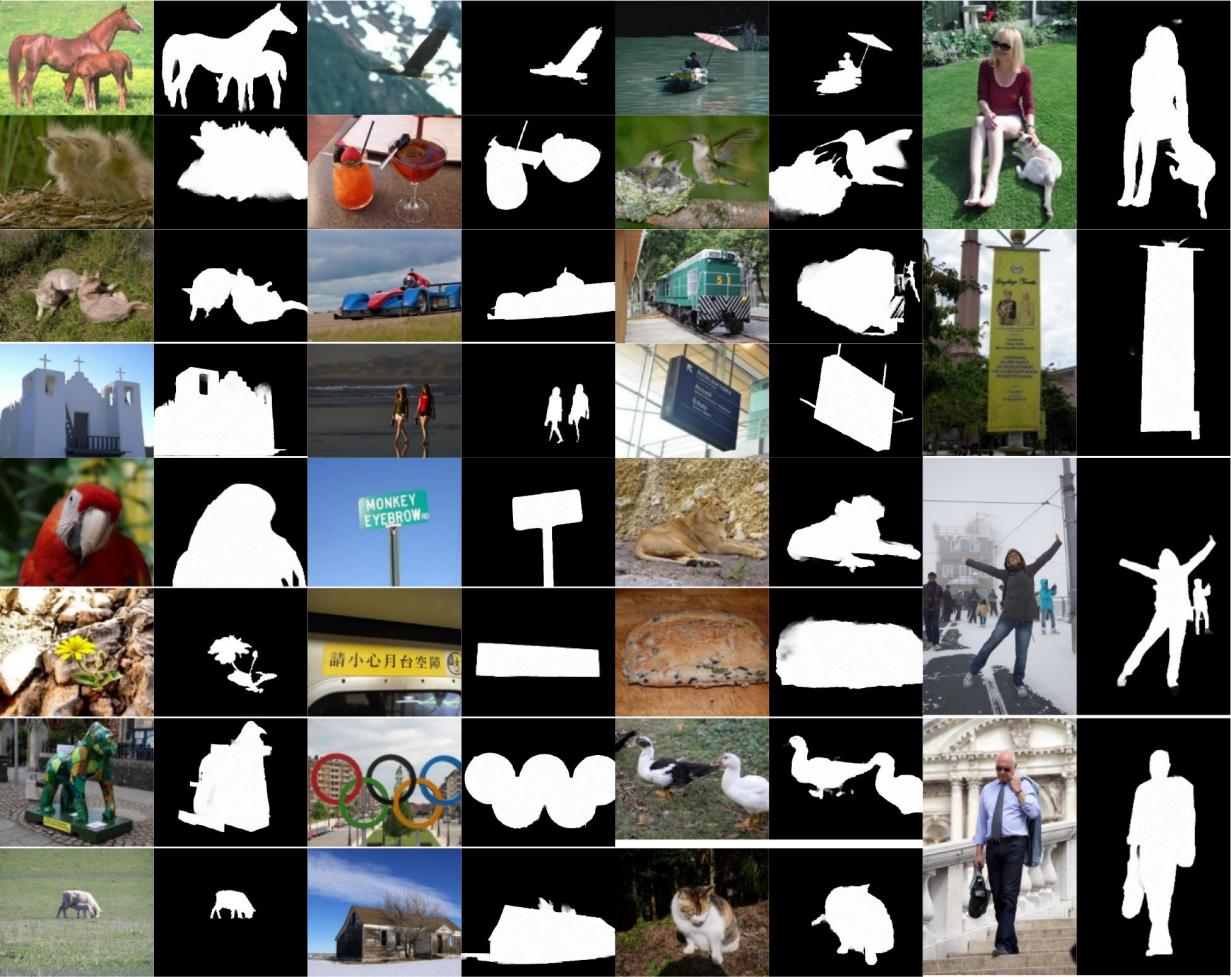
Original images and refined saliency mask (proposed method) pairs.

Figure 10 shows two sources of error due to Ground truth deficiency. The first source of error is that the ground truth includes only a part of the object rather than the entire object. This appears in Fig. 10, first and second rows. The second source is reflections and shadows of objects, where some Ground truth images consider reflections as salient objects, and others don't consider reflections as salient objects. Figure 10, the third row shows the second error source. Figure 11 shows more results for different sizes and number of objects, and low-contrast images in RGB-refined saliency mask pairs.

### Comparison with deep learning-based methods

Furthermore, the effectiveness of the refined Mask (proposed method) is evaluated against recent deep-learning approaches such as^54^, KSR^55^, BASNet^60^, EGNet^61^, CPD^62^, U2Net^63^, Method in^85^, PerGAN^86^, and GSCINet^87^. Figure 8 visually compares the proposed refined Mask (Fig. 8l) and deep-learning techniques (Fig. 8m–p). The proposed refined Mask produces outcomes comparable to those of deep-learning techniques.

As shown in Tables 1, 2, and 3, for ASD and MSRA10K datasets, the proposed refined Mask gives comparable and close results to deep learning methods. On the other hand, for the ECSSD dataset, the proposed refined Mask doesn't achieve the good results of deep-learning methods. Despite this, the results demonstrate notably better outcomes for the proposed method over some deep learning methods, specifically^54^ and KSR^55^. On the ECSSD dataset, the proposed method yields MAE values that are 3.1% and 0.3% lower than those obtained by^54^ and KSR^55^, respectively. For the MSRA10K dataset, the proposed method gives a 1.8% lower MAE value than the^54^ method.

Some deep-learning models for saliency detection may fail to preserve the object boundary and fine details due to network encoder-decoder architecture and the use of loss functions that don't consider the object edges and boundary. Also, these models introduce some inaccuracies related to scene interpretation, as the same object may be salient in some scenes and not salient in others. Moreover, deep-learning training is a highly computational step, needs high resources, is training-data dependent, and is time-consuming.

Therefore, incorporating the pre-trained CascadePSP, primarily designed for refining segmentation, into the background-priors saliency detection proves advantageous. This integration enhances the saliency mask refinement and preserves object boundaries by incorporating the Sobel operator into the loss function, eliminating the need to train complex networks. This encourages the adoption of hybrid models that leverage both pre-trained networks and background priors for the purpose of saliency detection.

## Conclusions

This paper presents a robust and effective method for detecting saliency within images. The proposed approach involves first refining the background dictionary to exclude salient object regions from the dictionary. Secondly, the dense and sparse representation reconstruction errors based on this dictionary are utilized as saliency values. Then, the generated saliency maps are post-processed and integrated to obtain the final saliency map. Finally, the salient detection mask is refined using the CascadePSP network. The experimental results demonstrate the superior performance of the proposed system compared to other methods, particularly in detecting near-boundary salient objects. The salient objects are uniformly highlighted, and the background is effectively suppressed. The results show the significant contribution of the refinement step using the CascadePSP network towards the accuracy and robustness of the saliency detection. In future studies, we will investigate the recent deep learning network to extract salient objects directly.

## Data Availability

The data generated and analyzed during the current study are available from the corresponding author on reasonable request. The ASD dataset is available online at https://ivrlwww.epfl.ch/supplementary_material/RK_CVPR09/. The ECSSD dataset is available online at http://www.cse.cuhk.edu.hk/leojia/projects/hsaliency/dataset.html. The MASR10K dataset is available online at https://mmcheng.net/msra10k/.
